# Impact of Empagliflozin on Glomerular Hyperfiltration and Albuminuria in Youth with Type 2 Diabetes: *Post Hoc* Analysis of DINAMO

**DOI:** 10.2215/CJN.0000000889

**Published:** 2025-11-11

**Authors:** Petter Bjornstad, Thomas Danne, Georgeanna J. Klingensmith, Elke Schueler, Igor Tartakovsky, Jan Marquard, Philip Zeitler, Steven Willi, Lori M. Laffel

**Affiliations:** 1 Seattle Children's Hospital, Seattle, Washington; 2 Auf der Bult Kinder- und Jugendkrankenhaus, Hannover, Germany; 3 Barbara Davis Center, University of Colorado, Aurora, Colorado; 4 mainanalytics GmbH, Sulzbach, Germany; 5 Boehringer Ingelheim International GmbH, Ingelheim, Germany; 6 Boehringer Ingelheim, Ridgefield, Connecticut; 7 Children's Hospital of Philadelphia, Philadelphia, Pennsylvania; 8 Joslin Diabetes Center, Harvard Medical School, Boston, Massachusetts

**Keywords:** albuminuria, diabetes, glomerular hyperfiltration

## Abstract

**Key Points:**

- We investigated the effects of empagliflozin on hyperfiltration and albuminuria in youth with type 2 diabetes.
- Hyperfiltration and albuminuria were significantly attenuated compared with placebo in adolescents aged 10–17 years with type 2 diabetes on empagliflozin.
- Additional studies are required to evaluate the effect of sodium-glucose cotransporter-2 inhibition on diabetic kidney disease risk in youth.

**Background:**

Hyperfiltration, common in youth with type 2 diabetes (T2D), may increase the risk of early diabetic kidney disease. The Diabetes Study of Linagliptin and Empagliflozin in Children and Adolescents and Diabetes Study of Linagliptin and Empagliflozin in Children and Adolescents MONO trials showed that compared with placebo, empagliflozin improved glycemic control in youth with T2D. This *post hoc* analysis evaluated empagliflozin versus placebo on selected parameters in participants from both trials according to their baseline (BL) hyperfiltration/normofiltration status.

**Methods:**

We calculated eGFR using the Zappitelli equation (combined serum creatinine and cystatin C), with hyperfiltration defined as 2 SDs above average for healthy youth per National Health and Nutrition Examination Survey (>126.8 ml/min per 1.73 m^2^). After randomization, 116 participants received empagliflozin (10 mg or 25 mg) or placebo. We compared responses to therapy for eGFR and urine albumin-creatinine ratio (UACR) at week 26 by BL hyperfiltration and normofiltration (≤126.8 ml/min per 1.73 m^2^), with empagliflozin groups pooled.

**Results:**

Empagliflozin treatment led to more significant reductions in eGFR among participants with BL hyperfiltration compared with normofiltration (*P*_interaction_ = 0.01). At week 26, eGFR decreased significantly with empagliflozin versus placebo in those with hyperfiltration (adjusted mean difference [95% confidence interval], −11.67 ml/min per 1.73 m^2^ [−19.90 to −3.43]; *P* = 0.006); 15% of those with hyperfiltration on empagliflozin shifted to normofiltration at week 26 versus 6% for placebo. At week 26, the geometric mean ratio of UACR was 55% lower with empagliflozin versus placebo in participants with UACR ≥30 mg/g at BL (0.45 [0.22 to 0.93]; *P* = 0.03). In the normofiltration subgroup, eGFR and UACR changes over time were similar between treatment groups.

**Conclusions:**

Empagliflozin was associated with attenuated hyperfiltration and albuminuria in youth with T2D. Future longitudinal evaluations can assess potential long-term benefits of treatment.

**Clinical Trial registry name and registration number:**

ClinicalTrials.gov, NCT03429543.

## Visual Abstract

**Figure:**
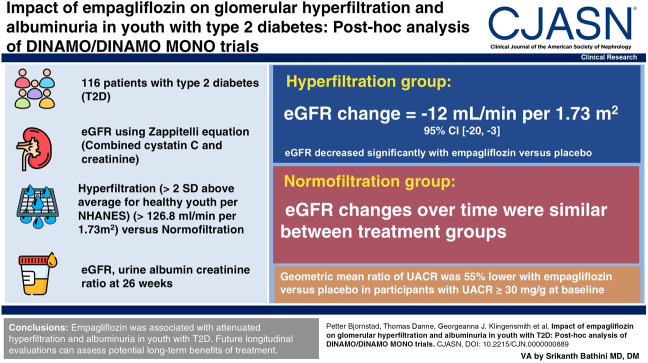

## Introduction

In young people, the prevalence of both type 2 diabetes (T2D) and diabetic kidney disease (DKD) is increasing globally. These surges have been paralleled by the worldwide epidemic of youth-onset obesity.^1,2^ The clinical course of T2D in adolescents appears more aggressive than in adults, with higher rates of intermediate and end-stage complications such as elevated albuminuria and kidney failure, respectively.^3^ In addition, adults with youth-onset T2D have evidence of more severe kidney structural lesions than those with adult-onset T2D, irrespective of diabetes duration.^4^ There are likely multiple factors that may contribute to the more aggressive course of youth-onset T2D, potentially related to the features of adolescent growth and development. For example, *post hoc* analysis of the Treatment Options for Type 2 Diabetes in Adolescents and Youth (TODAY) randomized clinical trial suggested that the growth hormone signaling mediators IGF-1, growth hormone receptor, and IGF binding protein-1 are associated with loss of glycemic control in youth-onset T2D.^5^ IGF-1 is known to mainly bind to IGF binding protein-3 (IGFBP-3),^6^ and both have been linked to kidney function, CKD, and DKD.^7–10^ DKD remains a leading cause of kidney failure, accounting for 44.5% of cases in developed countries.^11^ Despite the gravity of DKD, which highlights the need for optimal management of T2D early in its course, therapeutic options for youth-onset T2D are limited and often times unsatisfactory. For example, efficacy and safety data from pediatric clinical trials are currently lacking for several drugs approved for the treatment of adults with T2D.^12^ Furthermore, there are well-known self-management issues with the use of injectables (*e.g*., insulin and glucagon-like peptide-1 receptor agonists) by young people that lead to suboptimal utilization.^13^ As a result, there is an urgent need for improved and tolerable treatment options.

Both hyperfiltration, normally defined as a GFR >2 SDs above the mean GFR in healthy individuals,^14^ and moderate albuminuria are common in youth with T2D and may increase the risk of early DKD.^15,16^

The Diabetes Study of Linagliptin and Empagliflozin in Children and Adolescents (DINAMO) trial showed that compared with placebo, the sodium-glucose cotransporter-2 (SGLT2) inhibitor empagliflozin, an oral glucose-lowering drug, provided durable, clinically relevant improvements in glycemic control in youth with T2D aged 10–17 years.^17^ Its ancillary study, DINAMO MONO, investigated the same treatments in participants with T2D who were drug naïve or not on active therapy. It showed similar, albeit more modest, improvements in glycemic control with empagliflozin versus placebo.^18^ In contrast to studies in adults with T2D, there were no statistically significant differences in outcomes related to body weight or BP between those treated with empagliflozin and placebo in the DINAMO studies.^19^

The aim of this pooled *post hoc* analysis of the DINAMO and DINAMO MONO trials was to determine the effect of empagliflozin on various kidney-related outcomes, including eGFR and urine albumin-creatinine ratio (UACR), in youth with T2D according to baseline (BL) hyperfiltration or normofiltration status.

## Methods

### Study Population

The DINAMO and DINAMO MONO trials were operated under the same clinical trial protocol and statistical analysis plan. The results from both trials have been published previously.^17,18^ Briefly, the main inclusion criteria for both trials were participants aged 10–17 years at the time of randomization, with a documented diagnosis of T2D and insufficient glycemic control at screening (DINAMO: glycated hemoglobin [HbA1c] ≥6.5% and ≤10.5% [48–91 mmol/mol]; DINAMO MONO: HbA1c ≥6.5% and ≤9.0% [48–75 mmol/mol]). In DINAMO, participants were treated with diet and exercise plus background therapy with metformin and/or insulin. Participants in DINAMO MONO were drug naïve or not on active treatment before/at screening (including discontinuing metformin due to intolerance [or previous discontinuation for other reasons] and/or discontinuing insulin [total insulin use had to be ≤8 weeks] at the investigator's discretion).

Participants were randomized to either empagliflozin 10 mg or placebo once daily. After 12 weeks, participants randomized to empagliflozin 10 mg who did not attain HbA1c <7.0% (<53 mmol/mol) were rerandomized in a double-blinded manner at week 14 to either remain on empagliflozin 10 mg or uptitrate to 25 mg. Results for the two empagliflozin dosing groups were pooled.

### Data Collection

Blood and urine samples were collected at screening and BL visits, using standard assays, as reported previously.^17,18^ Hyperfiltration was defined as 2 SDs above the mean eGFR for healthy youth per National Health and Nutrition Examination Survey (>126.8 ml/min per 1.73 m^2^).^20^ eGFR was calculated using the Zappitelli combined creatinine and cystatin C prediction equation for GFR in children.^21^ Participants were grouped according to hyperfiltration (>126.8 ml/min per 1.73 m^2^) or normofiltration (≤126.8 ml/min per 1.73 m^2^) at BL.

### Statistical Analyses

BL characteristics for the subgroups were presented with descriptive statistics; categorical variables were compared using the chi-squared test and continuous variables using the *t* test. Responses to treatment were measured for changes in eGFR, UACR, systolic BP and diastolic BP (SBP and DBP, respectively), cystatin C, and creatinine over time from BL to week 26, with adjustment for BL values.

The effect of empagliflozin on changes in the parameters of interest over time was analyzed using a linear mixed model for repeated measures with adjustment for age (categorical), BL value (for the parameter of interest), visit, BL value-by-visit interaction, treatment-by-visit interaction, subgroup, treatment-by-subgroup interaction, visit-by-subgroup interaction, and treatment-by-visit-by-subgroup interaction. In a sensitivity analysis, eGFR was additionally analyzed using an extended model including adjustment for BL HbA1c and BL mean arterial pressure.

In addition, the proportion of participants with a change in glomerular filtration and UACR category (<30 versus ≥30 mg/g) from BL to week 26 was assessed descriptively using shift analyses. Safety was evaluated until week 26. Adverse events (AEs) of special interest included hypoglycemia, diabetic ketoacidosis, hepatic injury, and events leading to lower limb amputation. Preferred terms were reported according to the Medical Dictionary for Regulatory Activities, version 26.0.

All analyses were performed using SAS version 9.4 (SAS Institute, Cary, NC). All *P* values reported are two sided, and *P* < 0.05 was considered as statistically significant. No adjustments for multiple testing were made because of the exploratory nature of the study.

The trial that this *post hoc* analysis was based on was conducted in accordance with the principles of the Declaration of Helsinki and International Conference on Harmonization Good Clinical Practice guidelines and was approved by local authorities.^17^ Independent ethics committees/institutional review boards approved the protocol at participating centers. Participants provided written informed assent (youth) and consent (parents/guardians) before study entry.

## Results

### BL Characteristics

In total, 116 participants received either once-daily empagliflozin (*n*=58) or placebo (*n*=58). There was no difference in the distribution of normofiltration and hyperfiltration between the treatment groups, with a total of 61 (53%) and 55 (47%) participants, respectively (Table 1). When analyzed by trial, there was a similar distribution pattern of participants with normofiltration and hyperfiltration (DINAMO: 55 [52%] and 50 [48%], respectively; DINAMO MONO: 6 [55%] and 5 [46%], respectively).

**Table 1:** Baseline characteristics grouped by glomerular filtration status at baseline

Characteristic	Normofiltration (eGFR ≤126.8 ml/min per 1.73 m^2^)			Hyperfiltration (eGFR >126.8 ml/min per 1.73 m^2^)			Subgroup Comparison of the Two Total Columns
	Placebo (*n*=30)	Empa Pooled (*n*=31)	Total (*N*=61)	Placebo (*n*=28)	Empa Pooled (*n*=27)	Total (*N*=55)	
DINAMO	27 (90)	28 (90)	55 (90)	26 (93)	24 (89)	50 (91)	—
DINAMO MONO	3 (10)	3 (10)	6 (10)	2 (7)	3 (11)	5 (9)	—
Age, yr, mean (SD)	15 (2)	15 (2)	15 (2)	14 (2)	14 (2)	14 (2)	*P* = 0.27
Sex, male	15 (50)	11 (36)	26 (43)	6 (21)	8 (30)	14 (26)	*P* = 0.05
**Region**							*P* = 0.30
Asia	0 (0)	0 (0)	0 (0)	1 (4)	1 (4)	2 (34)	
Europe	4 (13)	5 (16)	9 (15)	4 (14)	1 (4)	5 (9)	
North America	20 (67)	20 (65)	40 (66)	18 (6)	22 (82)	40 (73)	
South America	6 (20)	6 (19)	12 (20)	5 (18)	3 (11)	8 (15)	
**Race^a^**							*P* = 0.04
American Indian or Alaska Native	2 (7)	5 (16)	7 (12)	0 (0)	2 (7)	2 (4)	
Asian	0 (0)	0 (0)	0 (0)	3 (11)	2 (7)	5 (9)	
Black	11 (37)	15 (48)	26 (43)	7 (25)	10 (37)	17 (31)	
Native Hawaiian or other Pacific Islander	1 (3)	0 (0)	1 (2)	0 (0)	0 (0)	0 (0)	
White	17 (57)	13 (42)	30 (49)	17 (61)	15 (56)	32 (58)	
**Ethnicity**							*P* = 0.54
Hispanic or Latino	12 (40)	8 (26)	20 (33)	11 (39)	10 (37)	21 (38)	
Not Hispanic or Latino	18 (60)	23 (74)	41 (67)	17 (61)	17 (63)	34 (62)	
**Time since diagnosis of T2D, yr**							*P* = 0.87
<1	12 (40)	11 (36)	23 (38)	10 (36)	10 (37)	20 (36)	
1–3	12 (40)	14 (45)	26 (43)	13 (46)	9 (33)	22 (40)	
>3	6 (20)	6 (19)	12 (20)	5 (18)	8 (30)	13 (24)	
Height, cm, mean (SD)	165.6 (12.1)	167.2 (10.1)	166.4 (11.1)	162.2 (8.8)	164.4 (9.8)	163.3 (9.3)	
Body weight, kg, mean (SD)	103.92 (25.40)	102.69 (26.29)	103.30 (25.65)	91.28 (30.92)	93.84 (21.52)	92.54 (26.51)	*P* = 0.03
BMI, kg/m^2^, mean (SD)	38.06 (9.42)	36.55 (8.39)	37.29 (8.87)	34.33 (10.17)	34.45 (5.86)	34.39 (8.26)	*P* = 0.07
**BMI *z* score**							*P* = 0.04
≤2 (underweight, normal, or overweight)	1 (3)	3 (10)	4 (7)	8 (29)	3 (11)	11 (20)	
>2 to ≤3 (class 1 obesity)	9 (30)	11 (36)	20 (33)	9 (32)	12 (44)	21 (38)	
>3 (class 2–3 obesity)	20 (67)	17 (55)	37 (61)	11 (39)	12 (44)	23 (42)	
eGFR, ml/min per 1.73 m^2^, mean (SD)	105 (12)	111 (10)	108 (11)	145 (16)	152 (22)	149 (19)	*P* < 0.001
**Tanner staging score**							*P* = 0.57
1	0 (0)	0 (0)	0 (0)	1 (4)	0 (0)	1 (12)	
2–4	12 (40)	13 (42)	25 (41)	10 (36)	12 (44)	22 (40)	
5	18 (60)	18 (58)	36 (59)	17 (61)	15 (56)	32 (58)	
IGF-1, nmol/L, mean (SD)	*n*=29 32.37 (13.38)	*n*=30 37.56 (10.46)	*N*=59 35.01 (12.17)	*n*=27 35.20 (14.84)	*n*=26 35.32 (13.86)	*N*=53 35.25 (14.23)	*P* = 0.92
IGFBP-3, nmol/L, mean (SD)	*n*=29 234.66 (50.92)	*n*=30 234.30 (45.43)	*N*=59 234.47 (47.79)	*n*=27 232.80 (62.61)	*n*=26 246.65 (64.01)	*N*=53 239.60 (63.08)	*P* = 0.63
**HbA1c, %**							
Mean (SD)	7.68 (1.35)	7.48 (1.07)	7.58 (1.21)	8.28 (1.05)	8.33 (1.38)	8.31 (1.21)	*P* = 0.002
<8.5%	23 (76.7)	26 (83.9)	49 (80.3)	18 (64.3)	16 (59.3)	34 (61.8)	*P* = 0.03
≥8.5%	7 (23.3)	5 (16.1)	12 (19.7)	10 (35.7)	11 (40.7)	21 (38.2)	
Fasting plasma glucose, mg/dl, mean (SD)	142.01 (57.11)	130.57 (49.47)	136.20 (53.23)	*n*=26 177.50 (65.61)	*n*=23 175.30 (55.64)	*N*=49 176.47 (60.52)	*P* < 0.001
Fasting C-peptide, nmol/L, mean (SD)	*n*=29 0.96 (0.45)	*n*=30 0.94 (0.37)	*N*=59 0.95 (0.41)	*n*=27 0.84 (0.39)	*n*=25 1.02 (0.68)	*N*=52 0.92 (0.55)	*P* = 0.79
**BP, mm Hg**							*P* = 0.92
SBP <140 and DBP <90	28 (93)	31 (100)	59 (97)	27 (96)	26 (96)	53 (96)	
SBP ≥140 or DBP ≥90	2 (7)	0 (0)	2 (3)	1 (4)	1 (4)	2 (4)	
**UACR, mg/g**							
Median (min–max)	*n*=29 7.69 (3.1–828.9)	*N*=29 7.81 (2.1–98.1)	*N*=58 7.75 (2.1–828.9)	*n*=27 10.53 (3.2–321.3)	*n*=26 15.25 (4.2–965.0)	*N*=53 12.43 (3.2–965.0)	*P* = 0.08^b^
<30	23 (77)	25 (81)	48 (79)	21 (75)	18 (67)	39 (71)	*P* = 0.47
30–300	4 (13)	4 (13)	8 (13)	5 (18)	7 (26)	12 (22)	
>300	2 (7)	0 (0)	2 (3)	1 (4)	1 (4)	2 (4)	
Missing	1 (3)	2 (7)	3 (5)	1 (4)	1 (4)	2 (4)	
**No. of background antidiabetic treatments**							*P* = 0.37
0	7 (23)	4 (13)	11 (18)	2 (7)	3 (11)	5 (9)	
1	13 (43)	16 (52)	29 (48)	17 (61)	13 (48)	30 (55)	
2	10 (33)	11 (36)	21 (34)	9 (32)	11 (41)	20 (36)	
**Background antidiabetic treatment**							*P* = 0.52
Metformin only	12 (40)	14 (45)	26 (43)	16 (57)	12 (44)	28 (51)	
Insulin only	1 (3)	2 (7)	3 (5)	1 (4)	1 (4)	2 (4)	
Metformin and insulin	10 (33)	11 (36)	21 (34)	9 (32)	11 (41)	20 (36)	
None (diet and exercise only, metformin not tolerated)	7 (23)	4 (13)	11 (18)	2 (7)	3 (11)	5 (9)	
**Metformin total daily dose, mg**							*P* = 0.56
Mean (SD)	*n*=22 1713.6 (737.8)	*n*=25 1634.0 (411.5)	*N*=47 1671.3 (581.8)	*n*=25 1722.0 (419.8)	*n*=23 1482.6 (548.3)	*N*=48 1607.3 (495.3)	
<1500	6 (20)	5 (16)	11 (18)	5 (18)	8 (30)	13 (24)	
≥1500	16 (53)	20 (65)	36 (59)	20 (71)	15 (56)	35 (64)	
No metformin	8 (27)	6 (19)	14 (23)	3 (11)	4 (15)	7 (13)	
Basal insulin total daily dose, IU/d, mean (SD)	*n*=11 49.7 (42.8)	*n*=13 42.1 (19.1)	*N*=24 45.6 (31.7)	*n*=10 55.2 (29.9)	*n*=12 78.7 (46.3)	*N*=22 68.0 (40.6)	*P* = 0.04
Basal insulin total daily dose, IU/d per kilogram, mean (SD)	*n*=11 0.53 (0.47)	*n*=13 0.47 (0.28)	*N*=24 0.50 (0.37)	*n*=10 0.63 (0.29)	*n*=12 0.89 (0.59)	*N*=22 0.77 (0.49)	*P* = 0.03

Data are *n* (%) unless stated otherwise. BMI, body mass index; DBP, diastolic BP; DINAMO, Diabetes Study of Linagliptin and Empagliflozin in Children and Adolescents; empa, empagliflozin; HbA1c, glycated hemoglobin; IGF-1, insulin-like growth factor-1; IGFBP-3, IGF binding protein-3; IU, International Unit; SBP, systolic BP; T2D, type 2 diabetes; UACR, urine albumin-creatinine ratio.

a More than one race category is possible for a participant.

b Based on log-transformed data.

Participants in both subgroups were of similar age and pubertal status, with no difference in levels of serum IGF-1 and IGFBP-3 (Table 1). However, compared with the normofiltration subgroup, the hyperfiltration subgroup comprised more commonly females (57% versus 75%, respectively) who were shorter (mean [SD] 166 [11] versus 163 [9] cm, respectively) and lighter (103.3 [25.7] versus 92.5 [26.5] kg, respectively). As a result, there was a higher proportion of participants with a body mass index *z* score ≤2 in the hyperfiltration versus normofiltration subgroup (20% versus 7%, respectively; Table 1).

Twice as many participants in the normofiltration group had no background antidiabetic therapy at BL (diet and exercise only, metformin not tolerated) versus the hyperfiltration subgroup (18% versus 9%, respectively). Compared with the normofiltration subgroup, the hyperfiltration subgroup had a higher mean (SD) basal insulin total daily dose per kilogram of body weight (0.50 [0.37] versus 0.77 [0.49] IU/d per kilogram, respectively), HbA1c (7.6 [1.2]% versus 8.3 [1.2]% [60 versus 67 mmol/mol], respectively), and fasting plasma glucose (136.2 [53.2] versus 176.5 [60.5] mg/dl, respectively), suggesting greater insulin resistance in the latter group (Table 1). In addition, compared with the normofiltration subgroup, moderate albuminuria (30–300 mg/g) was more common at BL in those with hyperfiltration (13% versus 22%, respectively; Table 1).

### Changes in Analyzed Parameters over Time

**Figure 1:**
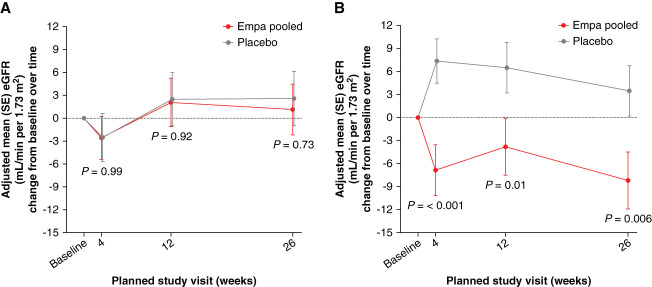
**MMRM results for change in eGFR from BL up to week 26 by BL filtration status**. (A) Normofiltration. (B) Hyperfiltration The *P* value for each study visit refers to the treatment difference in the eGFR change from BL for empa pooled versus placebo. The model included the effects of treatment, categorical age at randomization, BL eGFR, visit, BL eGFR-by-visit interaction, treatment-by-visit interaction, glomerular filtration, treatment-by-glomerular filtration interaction, visit-by-glomerular filtration interaction, and treatment-by-visit-by-glomerular filtration interaction. Normofiltration versus hyperfiltration *P*_interaction_ = 0.01. BL, baseline; empa, empagliflozin; MMRM, mixed model repeated measures.

Subgroup analysis according to hyperfiltration or normofiltration status at BL demonstrated that empagliflozin treatment led to greater reductions in eGFR in participants with hyperfiltration compared with those with normofiltration (*P*_interaction_ = 0.01; Figure 1). In the hyperfiltration subgroup, eGFR was significantly lower with empagliflozin versus placebo at all time points after BL (Figure 1B). At week 26, the adjusted mean difference (95% confidence interval [CI]) in eGFR was significantly lower for empagliflozin compared with placebo (−11.67 ml/min per 1.73 m^2^ [−19.90 to −3.43]; *P* = 0.006). By contrast, mean eGFR remained stable over the 26 weeks in the normofiltration subgroup in both treatment groups (Figure 1A). A sensitivity analysis including BL HbA1c and BL mean arterial pressure as factors had no effect on the results. Sensitivity analyses further adjusting for sex had no effect on the results. There were no significant changes between treatment groups from BL to week 26 for UACR, SBP, DBP, cystatin C, and creatinine (Supplemental Figures 1–5).

**Figure 2:**
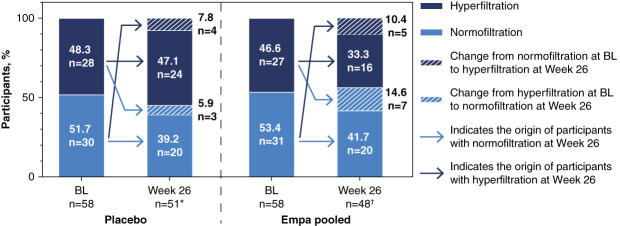
**Percentage of participants with a shift in glomerular filtration from BL to week 26.** *Week 26 data were missing for seven participants treated with placebo. †Week 26 data were missing for ten participants treated with empagliflozin. BL, baseline; empa, empagliflozin.

The shift analysis showed that 7 (26%) of 27 participants in the BL hyperfiltration subgroup treated with empagliflozin were restored to normofiltration at week 26, compared with only 3 (11%) of 28 participants in the BL hyperfiltration subgroup treated with placebo (Figure 2). In addition, 2 (8%) of the participants with moderate albuminuria and hyperfiltration at BL who were treated with empagliflozin shifted category to normoalbuminuria at week 26. One of these participants also reverted back to normofiltration, but the other participant withdrew from the study early and still had hyperfiltration at their last on-treatment value. This was in contrast to the placebo group, where no participants returned to normoalbuminuria, but 2 (8%) progressed to severe albuminuria at week 26 (Supplemental Figure 6).

When analyzed by BL UACR, there was a significantly lower UACR with empagliflozin versus placebo at all time points after BL in the subgroup of participants with BL UACR ≥30 mg/g (Supplemental Figure 7). At week 26, the geometric mean ratio (95% CI) of UACR was 55% lower with empagliflozin compared with placebo in participants with UACR ≥30 mg/g at BL (0.45 [0.22 to 0.93]; *P* = 0.03). Conversely, there was a parallel increase in both treatment groups from BL to week 26 in the subgroup with BL UACR <30 mg/g, with no notable difference between them.

The change in UACR over time was also examined by UACR (<30 and ≥30 mg/g) and hyperfiltration/normofiltration at BL. Participants on placebo in the hyperfiltration subgroup with UACR ≥30 mg/g at BL had a significant relative increase in UACR at week 26 compared with empagliflozin (geometric mean [95% CI], 5.79 mg/g [2.8 to 12.1] versus 0.93 mg/g [0.42 to 2.1], respectively; *P* < 0.001; Table 2). However, it is important to note that the participant numbers were very small (placebo, *N*=6; empagliflozin, *N*=5), and the UACR levels exhibited a high level of variance. In addition, in the hyperfiltration subgroup, there were more normoalbuminuric individuals receiving placebo than empagliflozin.

**Table 2:** Relative changes in urine albumin-creatinine ratio from baseline over time up to week 26 by glomerular filtration and baseline urine albumin-creatinine ratio

UACR	Normofiltration (eGFR ≤126.8 ml/min per 1.73 m^2^)				Hyperfiltration (eGFR >126.8 ml/min per 1.73 m^2^)			
	*n*	Adjusted Relative Change from BL	Comparison versus Placebo		*n*	Adjusted Relative Change from BL	Comparison versus Placebo	
		gMean (95% CI)	gMean Ratio (95% CI)	*P* Value		gMean (95% CI)	gMean Ratio (95% CI)	*P* Value
**UACR <30 mg/g**								
Placebo								
*Week 4*	21	1.13 (0.78 to 1.64)			19	1.14 (0.80 to 1.63)		
*Week 12*	21	1.09 (0.73 to 1.64)			19	1.06 (0.71 to 1.57)		
*Week 26*	19	1.00 (0.68 to 1.45)			20	1.13 (0.79 to 1.60)		
Empa pooled								
*Week 4*	22	1.17 (0.83 to 1.64)	1.03 (0.66 to 1.61)	0.90	17	1.04 (0.72 to 1.50)	0.92 (0.56 to 1.50)	0.72
*Week 12*	22	1.04 (0.72 to 1.52)	0.96 (0.58 to 1.56)	0.86	16	1.11 (0.74 to 1.68)	1.05 (0.61 to 1.82)	0.85
*Week 26*	20	0.97 (0.68 to 1.38)	0.97 (0.61 to 1.54)	0.91	17	1.34 (0.94 to 1.91)	1.19 (0.73 to 1.92)	0.48
**UACR ≥30 mg/g**								
Placebo								
*Week 4*	5	1.23 (0.51 to 2.96)			6	1.84 (0.89 to 3.79)		
*Week 12*	5	2.16 (0.82 to 5.68)			6	1.78 (0.80 to 3.94)		
*Week 26*	6	0.91 (0.39 to 2.13)			6	5.79 (2.77 to 12.07)		
Empa pooled								
*Week 4*	4	0.42 (0.19 to 0.94)	0.34 (0.12 to 0.95)	0.04	8	0.92 (0.46 to 1.85)	0.50 (0.23 to 1.12)	0.09
*Week 12*	4	0.99 (0.41 to 2.39)	0.46 (0.15 to 1.41)	0.17	6	0.85 (0.36 to 2.03)	0.48 (0.19 to 1.22)	0.12
*Week 26*	3	1.25 (0.50 to 3.13)	1.38 (0.48 to 3.92)	0.54	5	0.93 (0.42 to 2.06)	0.16 (0.07 to 0.38)	<0.001

BL, baseline; CI, confidence interval; empa, empagliflozin; gMean, geometric mean; UACR, urine albumin-creatinine ratio.

There was no significant correlation between relative changes in UACR and changes in eGFR from BL to week 26 for those with hyperfiltration receiving empagliflozin (*r*_s_=0.25; *P* = 0.26) or placebo (*r*_s_=0.24; *P* = 0.24) or for those with normofiltration receiving empagliflozin (*r*_s_=0.30; *P* = 0.17) or placebo (*r*_s_=−0.19; *P* = 0.38).

In the normofiltration subgroup, the change in HbA1c from BL up to week 26 was 0.49% for those treated with placebo and −0.12% for those treated with empagliflozin. In the hyperfiltration subgroup, this was 0.86% for placebo and −0.50% for empagliflozin.

### Safety

At week 26, AEs were reported in the normofiltration subgroup for 18 (60%) and 25 (81%) participants in the placebo and pooled empagliflozin groups, respectively (Supplemental Table 1). In the hyperfiltration subgroup, 20 (71%) and 21 (78%) participants reported AEs in the placebo and pooled empagliflozin groups, respectively. Serious AEs were reported in 1 (3%) and 2 (7%) participants on placebo and empagliflozin in the normofiltration subgroup, respectively, and one participant in each treatment group (placebo: 4%, empagliflozin: 4%) in the hyperfiltration subgroup (Supplemental Table 1).

Hypoglycemia was the most frequently reported AE. In both subgroups, the rate of hypoglycemic events was higher in participants treated with empagliflozin versus placebo (Supplemental Table 2). There was one event leading to lower limb amputation in a participant treated with empagliflozin in the normofiltration subgroup; this participant also had peripheral ischemia (Supplemental Table 2). The amputation was caused by peripheral ischemia (the participant had multiple thrombotic risk factors).

## Discussion

This pooled *post hoc* analysis of the DINAMO and DINAMO MONO trials demonstrated that reduction of eGFR with empagliflozin treatment was significantly greater in those with BL hyperfiltration compared with normofiltration. Empagliflozin therapy, compared with placebo, attenuated glomerular hyperfiltration in adolescents with T2D by reducing eGFR in individuals with hyperfiltration at all time points from BL through week 26. Furthermore, participants with BL albuminuria had significantly lower UACR levels with empagliflozin versus placebo throughout the 26 weeks, with a 55% lower geometric mean UACR ratio at week 26 relative to placebo, although it should be noted that numbers of participants within each subgroup were small; therefore, significance should be treated with caution.

Hyperfiltration was present in nearly half (47%) of the participants at BL. This is slightly higher than the reported rate of 25%–40% in the TODAY study of 10–17 year olds.^22^ Furthermore, moderate albuminuria at BL was present in over one fifth of participants in the hyperfiltration group. The TODAY study found that moderate albuminuria was only present in 6% of participants at BL.^23^ These two differences between the trials are probably a function of diabetes duration and glycemic control. The duration of T2D in the TODAY study was capped at <2 years, with a mean duration at entry of 7.8 months, whereas over 20% of participants in this study had durations of diabetes for over 3 years.^24^ Furthermore, HbA1c at randomization in the TODAY study was lower than in the DINAMO studies because the eligibility criteria required it to be <8.0%.^24^

Participants with hyperfiltration at BL had a higher mean HbA1c and basal insulin total daily dose per kilogram of body weight versus those with normofiltration, suggesting that hyperfiltration is associated with greater insulin resistance in this group. The TODAY study found a similar association, with lower estimated insulin sensitivity associated with the risk of hyperfiltration over time.^22^ Given this, the better glycemic response seen in those in the hyperfiltration subgroup receiving empagliflozin compared with normofiltration should be taken with caution because this could be driven by higher BL HbA1c. There were also a higher number of female participants in the hyperfiltration subgroup. This observation is consistent with previous studies showing a higher propensity for hyperfiltration in females with T2D compared with males.^25,26^ Furthermore, there were higher BL values of HbA1c for participants from DINAMO compared with those from DINAMO MONO, potentially owing to differing inclusion criteria for insufficient glycemic control at visit 1A (HbA1c ≥6.5% and ≤10.5% versus ≥6.5% and ≤9.0%). However, sensitivity analyses adjusting for BL HbA1c and sex did not affect the results.

Growth hormone and its mediator, IGF-1, have been linked to insulin resistance and have been thought to play a role in the development of diabetes-related complications in adults with T2D, but there are conflicting results in the published literature.^27,28^ In our study, there was no difference in mean concentrations of IGF-1 and its main circulating carrier, IGFBP-3, in the normofiltration and hyperfiltration subgroups at BL. This is consistent with the study by NeamŢu *et al.*, who found no relationship between the serum levels of IGF-1 and IGFBP-3 and GFR,^28^ but in contrast to the results of Lam *et al.*, who reported that IGF-1 was inversely correlated with GFR in their study of nearly 4000 adult patients from the Framingham Heart Study.^27^

Albuminuria was reduced in hyperfiltrating individuals but not in normofiltrating individuals. This suggests a hemodynamic effect of empagliflozin on albuminuria. In fact, lowering of intraglomerular pressure may explain the effect of empagliflozin on both UACR and eGFR in those with hyperfiltration. It has previously been demonstrated in young adults with type 1 diabetes that treatment with empagliflozin led to a reduction of eGFR in individuals with BL hyperfiltration, likely owing to afferent vasoconstriction and a decline in intraglomerular hypertension.^29^ This is also consistent with studies in adults with T2D where reduced intraglomerular hypertension results in an initial mean reduction in eGFR, a so-called eGFR dip, followed by stabilization of eGFR decline compared with placebo.^30–33^ It is important to note that a lack of attenuation of hyperfiltration in a subset of participants in this study does not necessarily imply the absence of long-term kidney protection. Trials have documented kidney protection in adult participants with T2D who experience both dipping versus nondipping GFRs in response to SGLT2 inhibition.^30,34^ In addition, Schaub *et al.* demonstrated that SGLT2 inhibition confers kidney protection in youth with T2D beyond its effects on tubuloglomerular feedback, including reversing perturbed kidney metabolism, a characteristic of diabetes.^35,36^

In this study, we observed increased hypoglycemic events in those treated with empagliflozin compared with placebo; however, it is worth noting that there were no severe hypoglycemic events. This could be because of a slightly higher number of participants receiving metformin plus insulin as background therapy, and the basal insulin total daily dose was higher on average for those receiving empagliflozin compared with placebo. These imbalances in background antidiabetic therapy may have contributed to the higher observation of hypoglycemia in those receiving empagliflozin because there is a known increased risk of hypoglycemia with insulin comedication with empagliflozin in adults.^37^

The strengths of this study include that it is the most comprehensive SGLT2 inhibitor trial in youth with T2D with kidney data and is one of the very few studies to examine the effects of this medication class on hyperfiltration and albuminuria. The study has several limitations worth mentioning. First, there are well-known challenges of clinical trial recruitment in pediatric patients with T2D.^38^ Furthermore, recruitment into DINAMO MONO was stopped prematurely on the basis of regulator feedback communicating a change of view on the value of a monotherapy pediatric trial and difficulties recruiting treatment-naïve participants;^18^ thus, both DINAMO trials had a relatively small sample size, which may affect the generalizability of the results. Second, this was an unspecified *post hoc* analysis of two trials of T2D in youth and of an exploratory nature. Furthermore, there may be residual confounding of results by background therapy and routine diet and exercise improvement efforts, which were interventions in the DINAMO trial. Third, only one urine sample was taken at each time point to measure UACR to minimize patient burden. It is worth noting that substantial variation in UACR is to be expected given the known biologic variability of UACR in patients with kidney disease.^39^ Finally, a substantial number of patients discontinued treatment before week 26, reducing the statistical power of the analyses. Nonetheless, these analyses offer important insight into the potential kidney protective effects of SGLT2 inhibition in youth with T2D.

In conclusion, the results of this *post hoc* analysis showed that empagliflozin treatment was clearly associated with attenuated hyperfiltration in youth with T2D. Although the potential long-term effects of such changes on DKD risk are unclear, these findings suggest a potential kidney protective role for empagliflozin in youth with T2D.

## Supplementary Material

## Data Availability

Original data generated for the study will be made available upon reasonable request to the corresponding author. Data Type: Clinical Trial Data. Reason for Restricted Access: To ensure independent interpretation of clinical study results and enable authors to fulfill their role and obligations under the International Committee of Medical Journal Editors criteria, Boehringer Ingelheim grants all external authors access to relevant clinical study data. In adherence with the Boehringer Ingelheim policy on transparency and publication of clinical study data, scientific and medical researchers can request access to clinical study data, typically 1 year after the approval has been granted by major regulatory authorities or after termination of the development program. Researchers should use the https://vivli.org/ link to request access to study data and visit https://www.mystudywindow.com/msw/datasharing for further information.

## Declarative Statements

This study is exempt from the Institutional Review Board or Ethics Committee approval, and I have provided the reason for exemption. This study reports *post hoc* analyses of clinical trial data. Approvals were given for the included studies. This study includes clinical experimentation and complies with the Declaration of Helsinki.

## Supplemental Material

Supplemental Table 1. Overall summary of AEs before week 26 treatment.

Supplemental Table 2. Overall number of AEs of special interest and other specific AEs before week 26 treatment.

Supplemental Figure 1. Relative changes in UACR from BL MMRM results over time up to week 26.

Supplemental Figure 2. Relative changes in SBP from BL MMRM results over time up to week 26.

Supplemental Figure 3. Relative changes in DBP from BL MMRM results over time up to week 26.

Supplemental Figure 4. Relative changes in Cystatin C from BL MMRM results over time up to week 26.

Supplemental Figure 5. Relative changes in Creatinine from BL MMRM results over time up to week 26.

Supplemental Figure 6. Percentage of participants with a shift in UACR from BL to the last on-treatment value up to week 26.

Supplemental Figure 7. Relative changes in UACR from BL MMRM results over time up to week 26 by BL UACR.
