# Supplementary material for: Impact of Empagliflozin on Glomerular Hyperfiltration and Albuminuria in Youth with Type 2 Diabetes: Post Hoc Analysis of DINAMO
Source: Clin J Am Soc Nephrol. Author manuscript; Available in PMC 2026 Feb 18. (PMC12889987; doi:10.2215/CJN.0000000889)
Supplement: Supplement [file NIHMS2131600-supplement-Supplement.pdf]

Supplemental Material

Supplemental Table 1. Overall summary of AEs prior to Week 26 treatment 2
Supplemental Table 2. Overall number of AESIs and other specific AEs prior to Week 26 treatment 3
Supplemental Figure 1. UACR relative change from baseline MMRM results over time up to Week 26 4
Supplemental Figure 2. SBP relative change from baseline MMRM results over time up to Week 26 5
Supplemental Figure 3. DBP relative change from baseline MMRM results over time up to Week 26 6
Supplemental Figure 4. Cystatin C relative change from baseline MMRM results over time up to Week 26 7
Supplemental Figure 5. Creatinine relative change from baseline MMRM results over time up to Week 26 8
Supplemental Figure 6. Percentage of participants with a shift in UACR from baseline to last value on-treatment up to Week 26 9
Supplemental Figure 7. UACR relative change from baseline MMRM results over time up to Week 26 by baseline UACR 10

Supplemental Table 1—Overall summary of AEs prior to Week 26 treatment

	Normofiltration (eGFR ≤126.8 mL/min per 1.73 m^2)		Hyperfiltration (eGFR >126.8 mL/min per 1.73 m^2)	
	**Placebo (*n* = 30)**	**Empa pooled (*n* = 31)***	**Placebo (*n* = 28)**	**Empa pooled (*n* = 27)**
Any AEs	18 (60.0)	25 (80.6)	20 (71.4)	21 (77.8)
Severe AEs	0	1 (3.2)	2 (7.1)	1 (3.7)
Drug-related AEs (investigator defined)	4 (13.3)	4 (12.9)	3 (10.7)	6 (22.2)
AEs leading to discontinuation	1 (3.3)	2 (6.5)	1 (3.6)	0
Serious AEs	1 (3.3)	2 (6.5)	1 (3.6)	1 (3.7)
Fatal	0	0	0	0
Life threatening	0	1 (3.2)	1 (3.6)	1 (3.7)
Persistent or significant disability or incapacity	0	1 (3.2)	0	0
Requiring or prolonging hospitalization	1 (3.3)	2 (6.5)	1 (3.6)	1 (3.7)
Congenital anomaly or birth defect	0	0	0	0
Other	0	0	0	1 (3.7)
Other significant AEs†	1 (3.3)	1 (3.2)	0	0

Data are n (%). AE, adverse event; eGFR, estimated glomerular filtration rate; empa, empagliflozin; ICH, International Council on Harmonisation.

*One participant had multiple thrombotic risk factors (heterozygosity of the factor V Leiden mutation, obesity, and use of systemic hormonal contraception).

†According to ICH E3.

Supplemental Table 2—Overall number of AESIs* and other specific AEs prior to Week 26 treatment

	Normofiltration (eGFR ≤126.8 mL/min per 1.73 m^2)		Hyperfiltration (eGFR >126.8 mL/min per 1.73 m^2)	
	**Placebo (*n* = 30)**	**Empa pooled (*n* = 31)†**	**Placebo (*n* = 28)**	**Empa pooled (*n* = 27)**
Hypersensitivity reactions	2 (6.7)	0	0	4 (14.8)
Pancreatitis	0	0	1 (3.6)	0
Hepatic injury	1 (3.3)	2 (6.5)	1 (3.6)	0
Decreased renal function	0	0	1 (3.6)	0
Diabetic ketoacidosis‡	0	0	1 (3.6)	0
Ketone measurements reported as an AE	1 (3.3)	1 (3.2)	1 (3.6)	1 (3.7)
Events leading to lower limb amputation‡	0	1 (3.2)	0	0
Peripheral ischemia	0	1 (3.2)	0	0
Hypoglycemia	3 (10.0)	6 (19.4)	4 (14.3)	6 (22.2)
Urinary tract infection‡	1 (3.3)	1 (3.2)	0	5 (18.5)
Genital infection‡	0	2 (6.5)	2 (7.1)	1 (3.7)
Arthralgia	0	1 (3.2)	1 (3.6)	0
Volume depletion	0	0	1 (3.6)	0
Acute pyelonephritis or urosepsis‡	0	0	0	0
Bone fracture	0	0	0	0
Pemphigoid in bullous conditions	0	0	0	0
Skin lesions	0	0	0	0

Data are n (%). AE, adverse event; AESI, AE of special interest; eGFR, estimated glomerular filtration rate; empa, empagliflozin; MedDRA, Medical Dictionary for Regulatory Activities.

*AESIs are defined by a list of preferred terms defined by standard or sponsor-defined MedDRA queries.

†One participant had multiple thrombotic risk factors (heterozygosity of the factor V Leiden mutation, obesity, and use of systemic hormonal contraception).

‡Investigator assessed/determined.

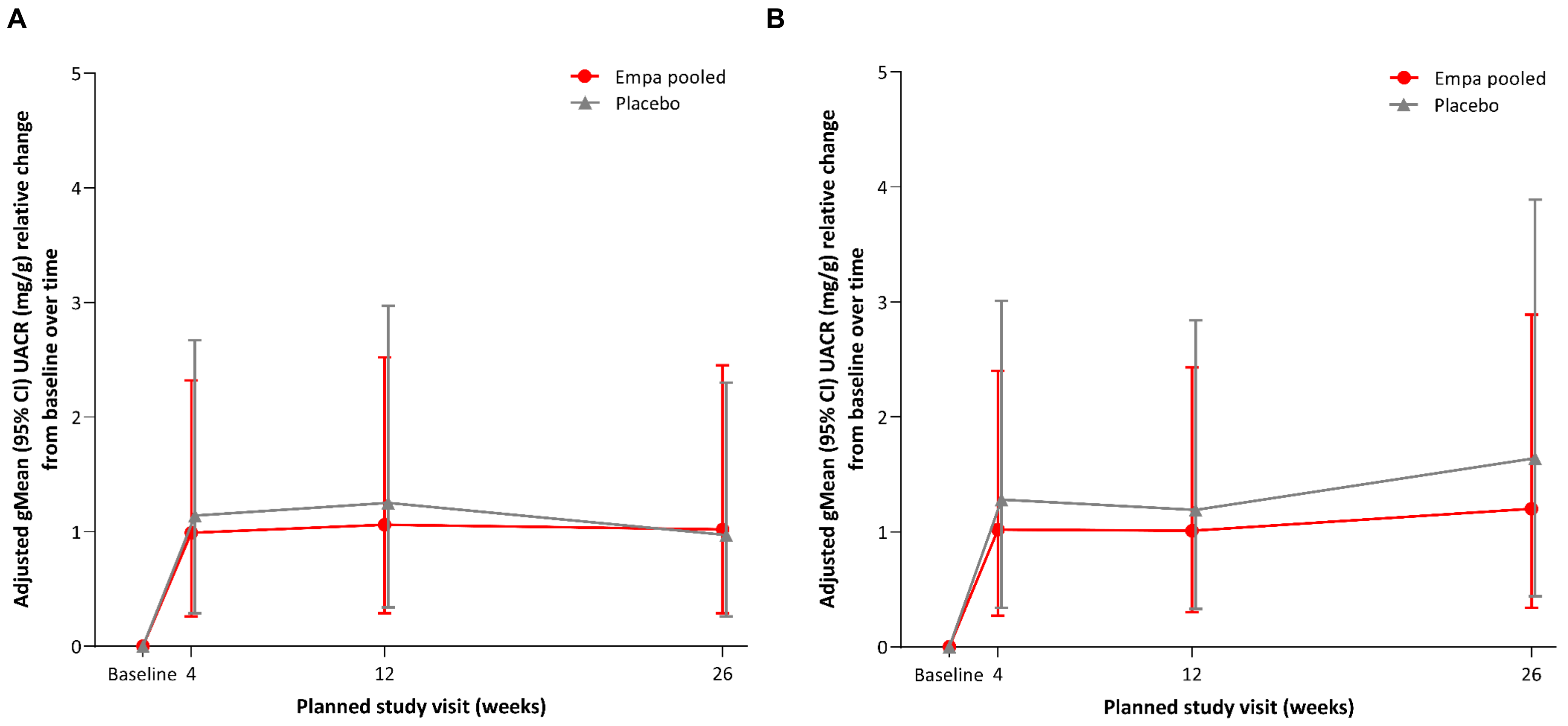

Supplemental Figure 1—UACR relative change from baseline MMRM results over time up to Week 26 by (*A*) normofiltration and (*B*) hyperfiltration at baseline. Normofiltration vs. hyperfiltration $P_{\text{interaction}}$ = 0.52.

Empa, empagliflozin; MMRM, mixed model repeated measures; UACR, urine albumin-to-creatinine ratio.

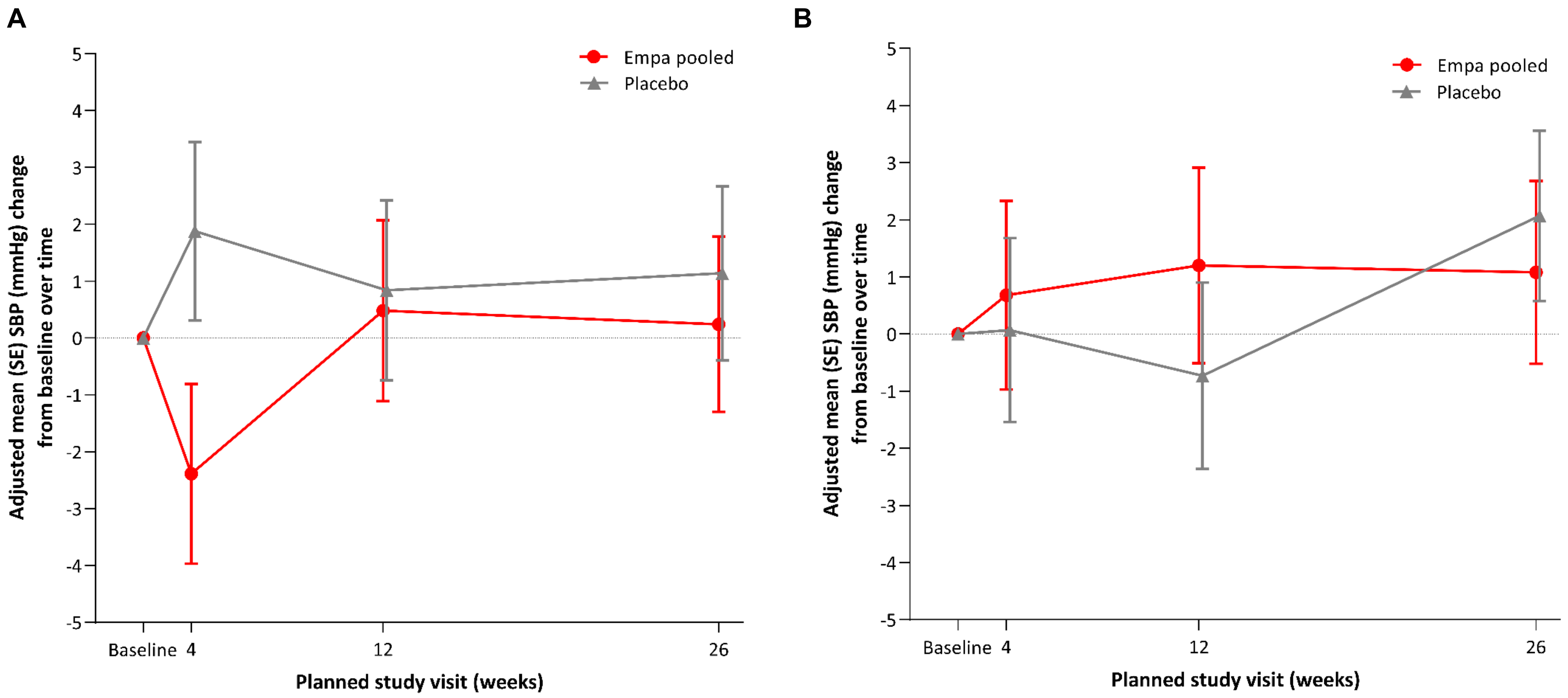

Supplemental Figure 2—SBP relative change from baseline MMRM results over time up to Week 26 by (*A*) normofiltration and (*B*) hyperfiltration at baseline. Normofiltration vs. hyperfiltration $P_{interaction}$ = 0.30.

Empa, empagliflozin; MMRM, mixed model repeated measures; SBP, systolic blood pressure.

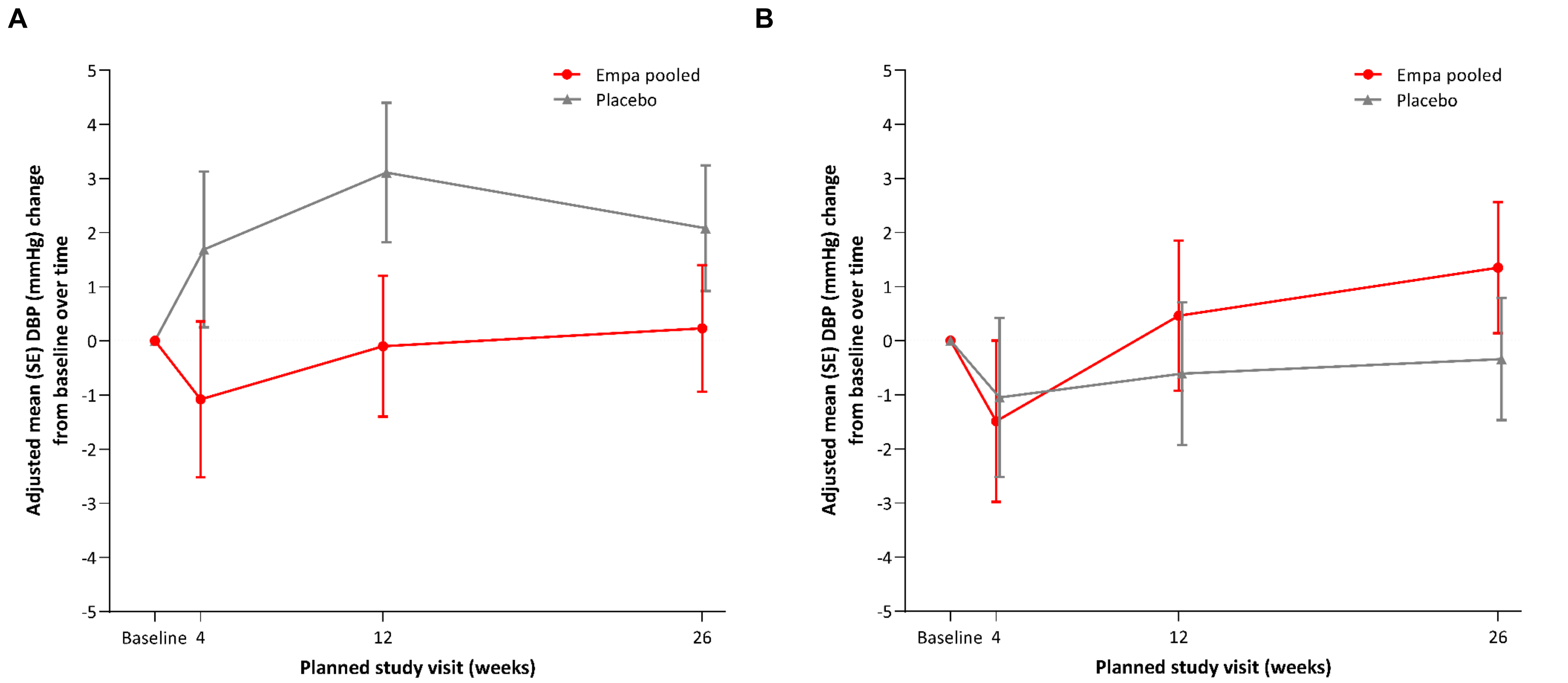

Supplemental Figure 3—DBP relative change from baseline MMRM results over time up to Week 26 by (*A*) normofiltration and (*B*) hyperfiltration at baseline. Normofiltration vs. hyperfiltration $P_{interaction}$ = 0.08.

DBP, diastolic blood pressure; empa, empagliflozin; MMRM, mixed model repeated measures.

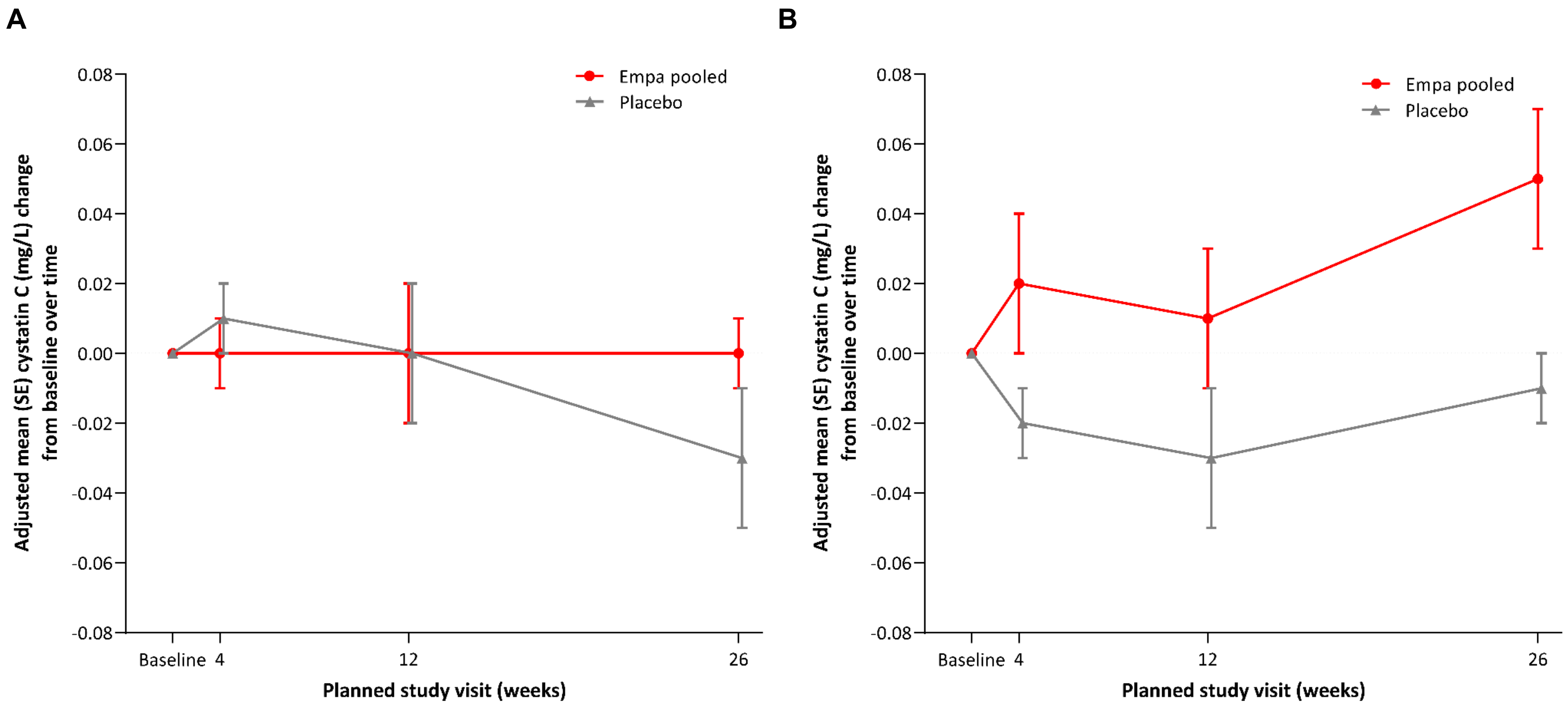

Supplemental Figure 4—Cystatin C relative change from baseline MMRM results over time up to Week 26 by (*A*) normofiltration and (*B*) hyperfiltration at baseline. Normofiltration vs. hyperfiltration $P_{\text{interaction}}$ = 0.09.

Empa, empagliflozin; MMRM, mixed model repeated measures.

A

B

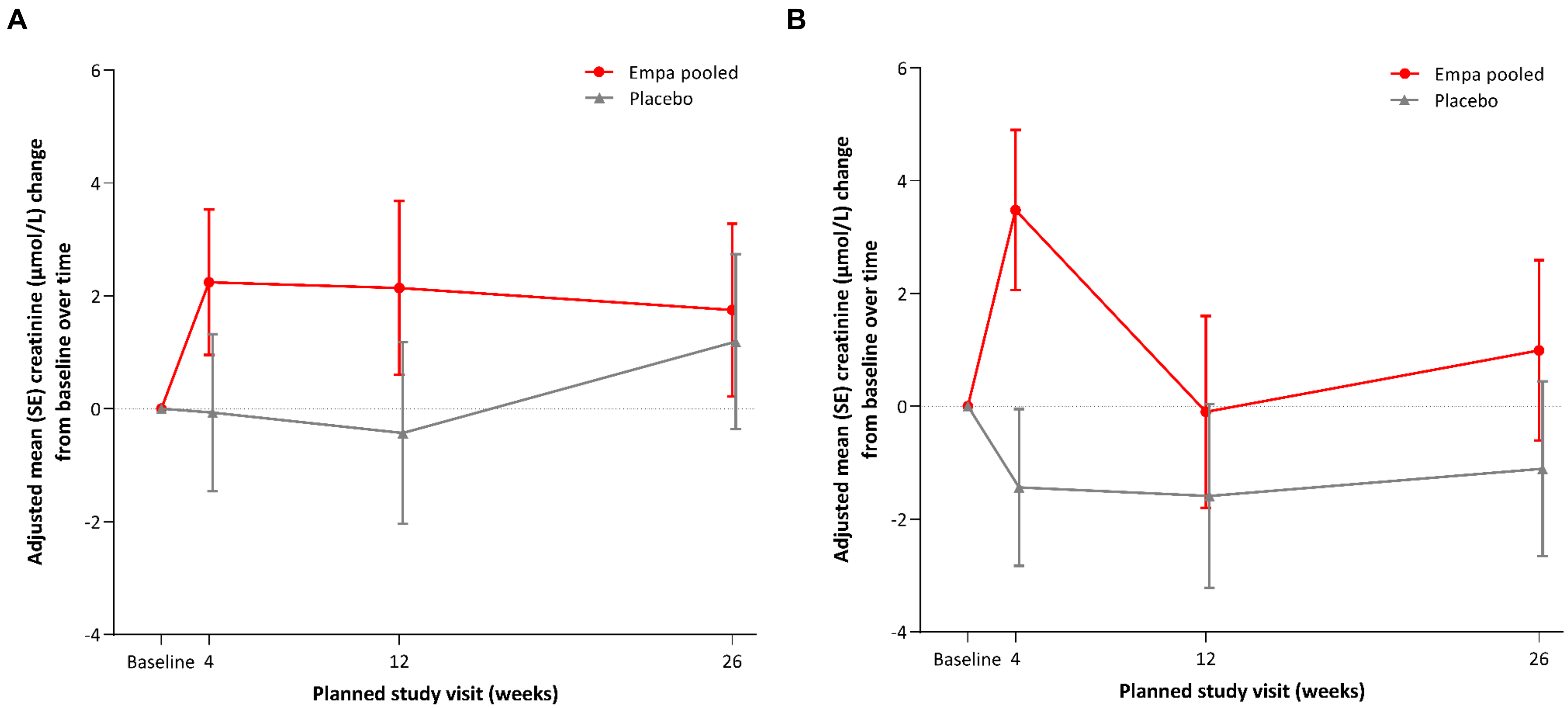

Supplemental Figure 5—Creatinine relative change from baseline MMRM results over time up to Week 26 by (*A*) normofiltration and (*B*) hyperfiltration at baseline. Normofiltration vs. hyperfiltration $P_{\text{interaction}}$ = 0.64.

Empa, empagliflozin; MMRM, mixed model repeated measures.

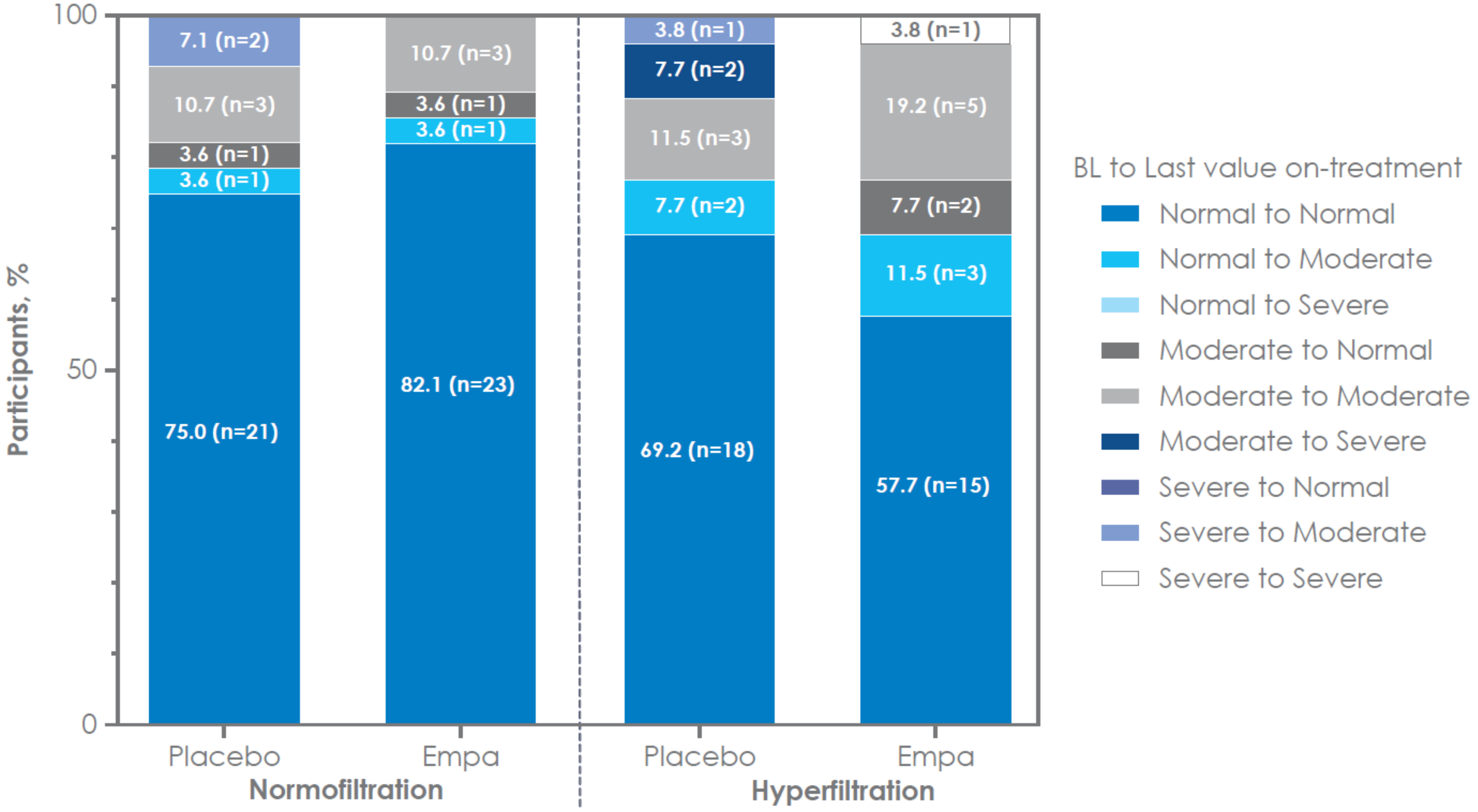

Supplemental Figure 6—Percentage of participants with a shift in albuminuria (UACR) from baseline to last value on-treatment up to Week 26. BL, baseline; empa, empagliflozin; UACR urine albumin-to-creatinine ratio.

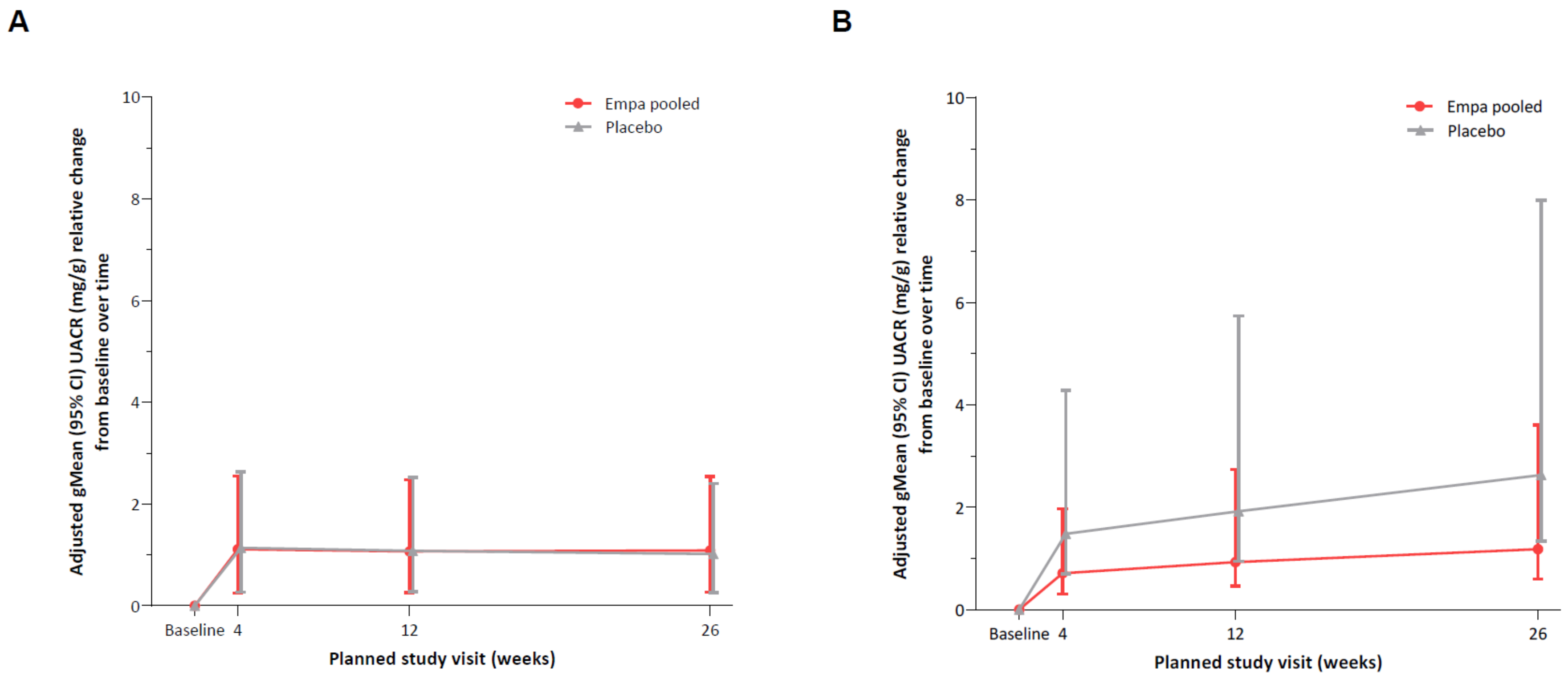

Supplemental Figure 7—UACR relative change from baseline MMRM results over time up to Week 26 by baseline UACR (*A*) <30 mg/g and (*B*) ≥30 mg/g. Empa, empagliflozin; MMRM, mixed model repeated measures; UACR, urine albumin-to-creatinine ratio.
