# Supplementary material for: Impact of Empagliflozin on Glomerular Hyperfiltration and Albuminuria in Youth with Type 2 Diabetes: Post Hoc Analysis of DINAMO
Source: Clin J Am Soc Nephrol. 2025 Nov 11;21(2):273–82. doi: 10.2215/CJN.0000000889 (PMC12889987; doi:10.2215/CJN.0000000889)
Supplement: Supplementary file 1 [file cjasn-21-273-s002.pdf]

ASN American Society of Nephrology
JASN Journal of the American Society of Nephrology
KIDNEY360 Accessing Our World From Every Angle
CJASN Clinical Journal of the American Society of Nephrology

ASN Journal Disclosure Form

As per ASN journal policy, I have disclosed any financial relationships or commitments I have held in the past 36 months as included below. I have listed my Current Employer below to indicate there is a relationship requiring disclosure. If no relationship exists, my Current Employer is not listed.

P. Bjornstad reports the following:
Employer: University of Washington; Consultancy: AstraZeneca; Boehringer Ingelheim; Bayer; Horizon Pharma; XORTX; Novo Nordisk; Lilly; Research Funding: Horizon Pharma/Amgen, AstraZeneca, Merck, Novo Nordisk, Lilly; and Advisory or Leadership Role: Horizon Pharma/Amgen, XORTX, AstraZeneca, Boehringer-Ingelheim, Bayer, Lilly, Novo Nordisk.

I understand that the information above will be published within the journal article, if accepted, and that failure to comply and/or to accurately and completely report the potential financial conflicts of interest could lead to the following: 1) Prior to publication, article rejection, or 2) Post-publication, sanctions ranging from, but not limited to, issuing a correction, reporting the inaccurate information to the authors' institution, banning authors from submitting work to ASN journals for varying lengths of time, and/or retraction of the published work.

Name: Petter Bjornstad
Manuscript ID: CJASN-2025-000179R3
Manuscript Title: Impact of empagliflozin on glomerular hyperfiltration and albuminuria in youth with T2D: Post-hoc analysis of DINAMO/DINAMO MONO trials
Date of Completion: September 6, 2025
Disclosure Updated Date: February 17, 2025

ASN American Society of Nephrology | JASN Journal of the American Society of Nephrology | KIDNEY360 Accessing Our World From Every Angle | CJASN Clinical Journal of the American Society of Nephrology

ASN Journal Disclosure Form

As per ASN journal policy, I have disclosed any financial relationships or commitments I have held in the past 36 months as included below. I have listed my Current Employer below to indicate there is a relationship requiring disclosure. If no relationship exists, my Current Employer is not listed.

T. Danne reports the following:
Employer: Auf der Bult Kinder und Jugendkrankenhaus; and Advisory or Leadership Role: Chief medical Officer of Breakthrough T1D (formerly JDRF).

I understand that the information above will be published within the journal article, if accepted, and that failure to comply and/or to accurately and completely report the potential financial conflicts of interest could lead to the following: 1) Prior to publication, article rejection, or 2) Post-publication, sanctions ranging from, but not limited to, issuing a correction, reporting the inaccurate information to the authors' institution, banning authors from submitting work to ASN journals for varying lengths of time, and/or retraction of the published work.

Name: Thomas Danne
Manuscript ID: CJASN-2025-000179R4
Manuscript Title: Impact of empagliflozin on glomerular hyperfiltration and albuminuria in youth with T2D: Post-hoc analysis of DINAMO
Date of Completion: October 3, 2025
Disclosure Updated Date: October 3, 2025

ASN American Society of Nephrology | JASN Journal of the American Society of Nephrology | KIDNEY360 Accessing Our World From Every Angle | CJASN Clinical Journal of the American Society of Nephrology

ASN Journal Disclosure Form

As per ASN journal policy, I have disclosed any financial relationships or commitments I have held in the past 36 months as included below. I have listed my Current Employer below to indicate there is a relationship requiring disclosure. If no relationship exists, my Current Employer is not listed.

G. Klingensmith reports the following:
Consultancy: Novo Nordisk; Boehringer Ingelheim; Takada; and Ownership Interest: Eli Lilly.

I understand that the information above will be published within the journal article, if accepted, and that failure to comply and/or to accurately and completely report the potential financial conflicts of interest could lead to the following: 1) Prior to publication, article rejection, or 2) Post-publication, sanctions ranging from, but not limited to, issuing a correction, reporting the inaccurate information to the authors' institution, banning authors from submitting work to ASN journals for varying lengths of time, and/or retraction of the published work.

Name: Georgeanna J Klingensmith
Manuscript ID: CJASN-2025-000179R3
Manuscript Title: "Impact of empagliflozin on glomerular hyperfiltration and albuminuria in youth with T2D: Post-hoc analysis of DINAMO/DINAMO MONO trials"
Date of Completion: September 3, 2025
Disclosure Updated Date: September 3, 2025

ASN
American Society of Nephrology
JASN
Journal of the American Society of Nephrology
KIDNEY360
Accessing Our World From Every Angle
CJASN
Clinical Journal of the American Society of Nephrology

ASN Journal Disclosure Form

As per ASN journal policy, I have disclosed any financial relationships or commitments I have held in the past 36 months as included below. I have listed my Current Employer below to indicate there is a relationship requiring disclosure. If no relationship exists, my Current Employer is not listed.

L. Laffel reports the following:
Consultancy: Boehringer Ingelheim, Janssen, Dexcom, Medtronic, Sequel, Tandem Diabetes, Sanofi, Mannkind, Arbor Biotech, Vertex;; Research Funding: Lilly, Medtronic, Insulet, Tandem Diabetes, Sequel Med Tech, Sinocare, Mannkind, Dexcom; and Honoraria: Dexcom, Insulet, Medtronic, Boehringer Ingelheim.

I understand that the information above will be published within the journal article, if accepted, and that failure to comply and/or to accurately and completely report the potential financial conflicts of interest could lead to the following: 1) Prior to publication, article rejection, or 2) Post-publication, sanctions ranging from, but not limited to, issuing a correction, reporting the inaccurate information to the authors' institution, banning authors from submitting work to ASN journals for varying lengths of time, and/or retraction of the published work.

Name: Lori M. Laffel
Manuscript ID: CJASN-2025-000179R3
Manuscript Title: Impact of empagliflozin on glomerular hyperfiltration and albuminuria in youth with T2D: Post-hoc analysis of DINAMO/DINAMO MONO trials
Date of Completion: September 6, 2025
Disclosure Updated Date: September 6, 2025

ASN American Society of Nephrology
JASN Journal of the American Society of Nephrology
KIDNEY360 Accessing Our World From Every Angle
CJASN Clinical Journal of the American Society of Nephrology

ASN Journal Disclosure Form

As per ASN journal policy, I have disclosed any financial relationships or commitments I have held in the past 36 months as included below. I have listed my Current Employer below to indicate there is a relationship requiring disclosure. If no relationship exists, my Current Employer is not listed.

J. Marquard reports the following:
Employer: Boehringer Ingelheim; Amgen; Merck & Co; and Ownership Interest: Merck @ Co.

I understand that the information above will be published within the journal article, if accepted, and that failure to comply and/or to accurately and completely report the potential financial conflicts of interest could lead to the following: 1) Prior to publication, article rejection, or 2) Post-publication, sanctions ranging from, but not limited to, issuing a correction, reporting the inaccurate information to the authors' institution, banning authors from submitting work to ASN journals for varying lengths of time, and/or retraction of the published work.

Name: Jan Marquard
Manuscript ID: CJASN-2025-000179R3
Manuscript Title: Impact of empagliflozin on glomerular hyperfiltration and albuminuria in youth with T2D: Post-hoc analysis of DINAMO/DINAMO MONO trials
Date of Completion: September 4, 2025
Disclosure Updated Date: September 4, 2025

ASN
American Society of Nephrology
JASN
Journal of the American Society of Nephrology
KIDNEY360
Accessing Our World From Every Angle
CJASN
Clinical Journal of the American Society of Nephrology

ASN Journal Disclosure Form

As per ASN journal policy, I have disclosed any financial relationships or commitments I have held in the past 36 months as included below. I have listed my Current Employer below to indicate there is a relationship requiring disclosure. If no relationship exists, my Current Employer is not listed.

P. Zeitler reports the following:
Employer: University of Colorado; and Consultancy: NovoNordisk.

I understand that the information above will be published within the journal article, if accepted, and that failure to comply and/or to accurately and completely report the potential financial conflicts of interest could lead to the following: 1) Prior to publication, article rejection, or 2) Post-publication, sanctions ranging from, but not limited to, issuing a correction, reporting the inaccurate information to the authors' institution, banning authors from submitting work to ASN journals for varying lengths of time, and/or retraction of the published work.

Name: Phil S Zeitler
Manuscript ID: CJASN-2025-000179R4
Manuscript Title: Impact of empagliflozin on glomerular hyperfiltration and albuminuria in youth with type 2 diabetes: Post-hoc analysis of DINAMO
Date of Completion: October 26, 2025
Disclosure Updated Date: October 26, 2025

ASN American Society of Nephrology | JASN Journal of the American Society of Nephrology | KIDNEY360 Accessing Our World From Every Angle | CJASN Clinical Journal of the American Society of Nephrology

ASN Journal Disclosure Form

As per ASN journal policy, I have disclosed any financial relationships or commitments I have held in the past 36 months as included below. I have listed my Current Employer below to indicate there is a relationship requiring disclosure. If no relationship exists, my Current Employer is not listed.

E. Schueler reports the following:
Employer: mainanalytics GmbH; and Ownership Interest: Merck KG; Sanofi.

I understand that the information above will be published within the journal article, if accepted, and that failure to comply and/or to accurately and completely report the potential financial conflicts of interest could lead to the following: 1) Prior to publication, article rejection, or 2) Post-publication, sanctions ranging from, but not limited to, issuing a correction, reporting the inaccurate information to the authors' institution, banning authors from submitting work to ASN journals for varying lengths of time, and/or retraction of the published work.

Name: Elke Schueler
Manuscript ID: CJASN-2025-000179R3
Manuscript Title: Impact of empagliflozin on glomerular hyperfiltration and albuminuria in youth with T2D: Post-hoc analysis of DINAMO/DINAMO MONO trials
Date of Completion: September 19, 2025
Disclosure Updated Date: September 19, 2025

ASN American Society of Nephrology | JASN Journal of the American Society of Nephrology | KIDNEY360 Accessing Our World From Every Angle | CJASN Clinical Journal of the American Society of Nephrology

ASN Journal Disclosure Form

As per ASN journal policy, I have disclosed any financial relationships or commitments I have held in the past 36 months as included below. I have listed my Current Employer below to indicate there is a relationship requiring disclosure. If no relationship exists, my Current Employer is not listed.

I. Tartakowsky reports the following:
Employer: Boehringer Ingelheim International GmbH

I understand that the information above will be published within the journal article, if accepted, and that failure to comply and/or to accurately and completely report the potential financial conflicts of interest could lead to the following: 1) Prior to publication, article rejection, or 2) Post-publication, sanctions ranging from, but not limited to, issuing a correction, reporting the inaccurate information to the authors' institution, banning authors from submitting work to ASN journals for varying lengths of time, and/or retraction of the published work.

Name: Igor Tartakowsky
Manuscript ID: CJASN-2025-000179R2
Manuscript Title: Impact of empagliflozin on glomerular hyperfiltration and albuminuria in youth with type 2 diabetes: Post-hoc analysis of DINAMO
Date of Completion: October 2, 2025
Disclosure Updated Date: October 2, 2025

ASN American Society of Nephrology
JASN Journal of the American Society of Nephrology
KIDNEY360 Accessing Our World From Every Angle
CJASN Clinical Journal of the American Society of Nephrology

ASN Journal Disclosure Form

As per ASN journal policy, I have disclosed any financial relationships or commitments I have held in the past 36 months as included below. I have listed my Current Employer below to indicate there is a relationship requiring disclosure. If no relationship exists, my Current Employer is not listed.

S. Willi reports the following:
Employer: Children's Hospital of Philadelphia; Consultancy: MannKind Corporation; Boehringer Ingelheim International GmbH; and Research Funding: SANOFI US SERVICES INC; Mannkind Corporation; Eli Lilly and Company.

I understand that the information above will be published within the journal article, if accepted, and that failure to comply and/or to accurately and completely report the potential financial conflicts of interest could lead to the following: 1) Prior to publication, article rejection, or 2) Post-publication, sanctions ranging from, but not limited to, issuing a correction, reporting the inaccurate information to the authors' institution, banning authors from submitting work to ASN journals for varying lengths of time, and/or retraction of the published work.

Name: Steve Willi
Manuscript ID: CJASN-2025-000179R3
Manuscript Title: Impact of empagliflozin on glomerular hyperfiltration and albuminuria in youth with T2D: Post-hoc analysis of DINAMO/DINAMO MONO trials
Date of Completion: September 3, 2025
Disclosure Updated Date: September 3, 2025
